# IFT80 Improves Invasion Ability in Gastric Cancer Cell Line via ift80/p75NGFR/MMP9 Signaling

**DOI:** 10.3390/ijms19113616

**Published:** 2018-11-16

**Authors:** Rui Wang, Xiaoyan Deng, Chengfu Yuan, Hongmei Xin, Geli Liu, Yong Zhu, Xue Jiang, Changdong Wang

**Affiliations:** 1 Department of Biochemistry and Molecular Biology, Molecular Medicine and Cancer Research Center, Chongqing Medical University, Chongqing 400016, China; cherrywangrui@163.com (R.W.); xydeng_cqmu@126.com (X.D.); 18369610590@163.com (H.X.); geli0907@sina.com (G.L.); 2 College of Medical Science, China Three Gorges University, Yichang 443002, China; Yuancf46@ctgu.edu.cn; 3 Department of Microbiology and Molecular Genetics, School of Medicine, University of California Irvine, Irvine, CA 92697, USA; yongz4@uci.edu

**Keywords:** intraflagellar transport, p75NGFR, MMP9, invasion, gastric cancer

## Abstract

The assembly and maintenance of cilia depend on intraflagellar transport (IFT) proteins, which play an important role in development and homeostasis. IFT80 is a newly defined IFT protein and partial mutation of IFT80 in humans causes diseases such as Jeune asphyxiating thoracic dystrophy (JATD) and short rib polydactyly (SRP) type III, both characterized by abnormal skeletal development. However, the role and mechanism of IFT80 in the invasion of gastric cancer is unknown. We established SGC-7901 and MKN-45 gastric cancer cell lines that stably overexpressed IFT80, as verified by quantitative reverse transcription-PCR, Western blot, and immunofluorescence. Matrix metalloproteinase-9 (MMP9) plays an important role in tumor invasion, and its expression was assessed by quantitative reverse transcription-PCR, Western blotting, and immunofluorescence. The invasion ability of IFT80 on SGC-7901 and MKN-45 cells was examined by the Matrigel invasion assay. The relationship between p75NGFR, and the p75NGFR antagonists, PD90780 and IFT80, were detected by quantitative reverse transcription-PCR and Western blotting. We first detected an IFT80 expression pattern, and found that IFT80 was highly expressed in gastric cancer clinical samples. Overexpression of IFT80 in the gastric cancer cell lines, SGC-7901 and MKN-45, led to lengthening cilia. Additionally, overexpression of IFT80 significantly improved proliferation and invasion, but inhibited apoptosis, in gastric cancer cells. We further found that overexpression of IFT80 increased p75NGFR and MMP9 mRNA and protein expression. Treatment with the p75NGFR antagonist PD90780 inhibited the increased invasion ability resulting from overexpression of IFT80 in SGC-7901 and MKN-45 gastric cancer cells. Thus, these results suggest that IFT80 plays an important role in invasion of gastric cancer through regulating the ift80/p75NGFR/MMP9 signal pathways.

## 1. Introduction

Gastric cancer is a common malignancy worldwide [1]. China has a high incidence of gastric cancer, and the number of deaths account for about 50% of the world’s total [2]. Currently, the 5-year survival rate of gastric cancer patients is less than 20%, due to the recurrence and metastasis of gastric cancer [3]. Although the treatment of gastric cancer has made great progress, its recurrence and metastasis often lead to treatment failure and death of the patients [4,5]. Although traditional clinicopathological factors, such as gastric cancer grading, staging, and lymph node metastasis, have been widely used to predict the recurrence and metastasis of gastric cancer, early prediction of gastric cancer survival and prognosis, and exploration of new biomarkers to guide clinical treatment, are necessary. The primary cilium was previously considered vestigial—assumed to be of little clinical significance. Primary cilia contain extracellular receptors, which are able to respond to chemosensation, mechanical stimulation and, in specialized cases, light, gravity, and temperature, as well as to a number of signaling pathways that are important in development and tissue homeostasis [6,7]. Cilia are microtubule-based structures that protrude from almost all eukaryotic cells, including photoreceptors, which play essential roles in vertebrate development, signaling, cellular motility, sensory transduction, and homeostasis [8,9]. Cilia formation and function requires effective intraflagellar transport (IFT), which plays an important role in development and in vivo balance. Accumulating evidence has certified that defects in some IFT proteins, such as IFT88 [10] and IFT172 [11], result in impaired cilia formation. 

IFT80 is a newly defined IFT protein [12]. The key proteins of cilia are IFT proteins, with more than 20 proteins divided into IFT-A and IFT-B complexes [13]. IFT80, a protein component of IFT complex B, is required for the formation, maintenance, and functionality of cilia [14,15]. Mutations in IFT80 causes Jeune asphyxiating thoracic dystrophy (JATD) and short rib polydactyly (SRP) type III [16]. Both diseases are characterized by bone abnormalities, including constriction of the ribcage, polydactyly, and shortening of the long bones [16,17,18,19]. In addition, the Hedgehog (Hh) signal transduced by IFT proteins is essential for osteoblast differentiation and bone development [12,20]. At the molecular level, the Hedgehog signaling pathway is found to be abnormally active in various types of cancers [21]. As defects in an IFT protein can cause abnormalities in the Hedgehog signal, IFT proteins can be related to cancer. However, whether or not the IFT80 protein is associated with gastric cancer cell proliferation, metastatic infiltration, and cell apoptosis, is not clear.

Matrix metallopeptidases (MMPs) are one important group of proteolytic enzymes that are capable of degrading components of the extracellular matrix and basement membrane [22]. Many human tumors have been shown to be associated with increased expression of MMP2 and MMP9 [23]. MMP2 and MMP9 have also been reported to be correlated with poor survival in ovarian cancer patients and promoted ovarian cancer cell invasion [24]. Invasion and metastatic disease represent the underlying cause of morbidity and mortality for most solid tumors [25,26]. In this study, we evaluated the expression of MMP9 in gastric cancer cell lines, and studied its effect on gastric cancer cell invasion. Our results indicated that IFT80 enhanced the invasion ability in gastric cancer via upregulation of MMP9 protein expression.

## 2. Results

### 2.1. IFT80 Is Highly Expressed in Clinical Gastric Cancer

**Figure 1:**
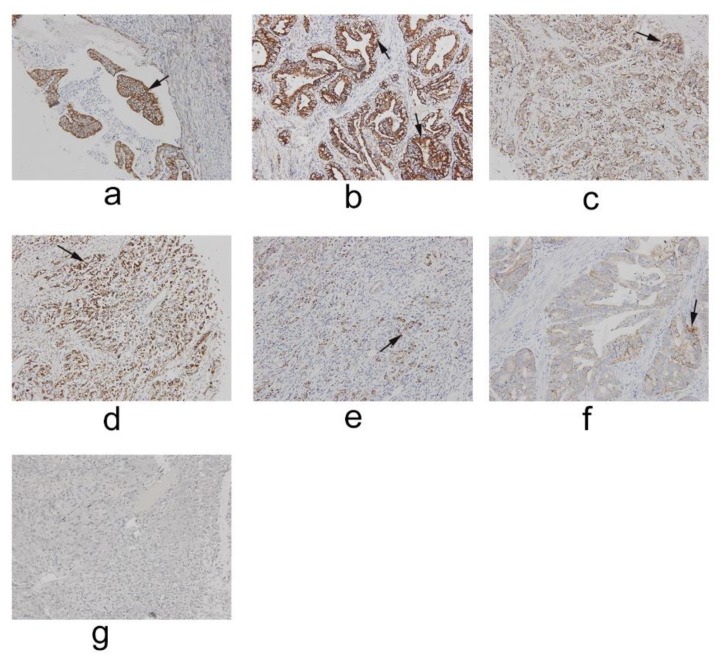
Immunohistochemical analysis of IFT80 in different types of gastric cancer. Expression of IFT80 was determined by immunohistochemical staining with an IFT80 antibody. (**a**) Gastric cancer tissue (type: malignant; pathology: adenocarcinoma; stage: II; TNM stage: T3N0M0; grade: 1) (200×). (**b**) Gastric cancer tissue (type: malignant; pathology: adenocarcinoma; stage: II; TNM stage: T3N0M0; grade: 2) (200×). (**c**) Gastric cancer tissue (type: malignant; pathology: adenocarcinoma; stage: IIIa; TNM stage: T3N1M0; grade: 3) (200×). (**d**) Gastric cancer tissue (type: malignant; pathology: adenocarcinoma; stage: IV; TNM stage: T3N3M0; grade: 3) (200×). (**e**) Gastric cancer tissue (type: malignant; pathology: adenocarcinoma; stage: IV; TNM stage: T3N3M0; grade: 3) (200×). (**f**) Gastric cancer tissue (type: malignant; pathology: papillary adenocarcinoma; stage: II; TNM stage: T3N0M0; grade: 2) (200×). (**g**) Normal gastric tissue (200×).

To identify the expression pattern of IFT80, we first analyzed IFT80 expression in 48 clinical gastric cancer tissues by immunohistochemistry. The results showed that IFT80 was highly expressed in clinical gastric cancer. IFT80 expression was found in higher levels in gastric cancer tumor tissue than in normal gastric tissue (Figure 1, indicated by the arrow).

### 2.2. Overexpression of IFT80 Increases IFT80 Expression and Improves Cilia Formation in the SGC-7901 Gastric Cancer Cell Line

**Figure 2:**
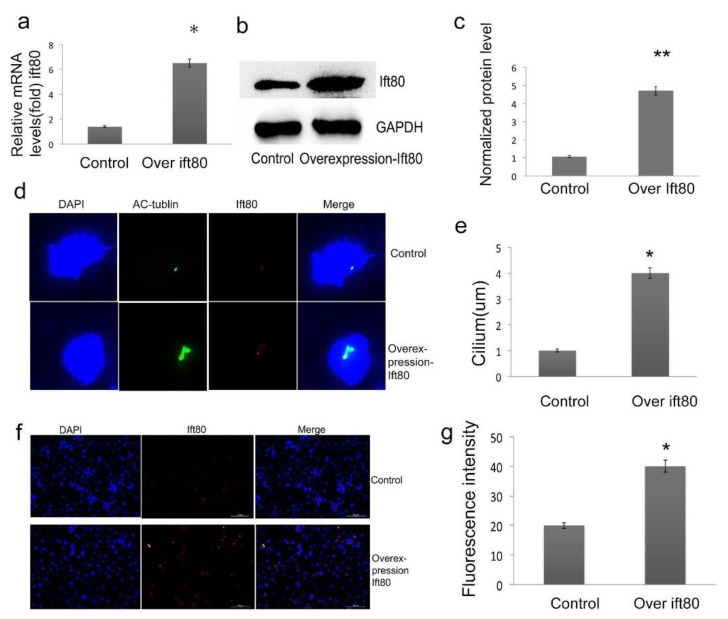
Overexpression of IFT80 in SGC-7901 gastric carcinoma cells. (**a**) Representative images of mRNA levels from the RT-PCR assay. (**b**) After stable transfection, the overexpression of IFT80 was evaluated by Western blot. GAPDH served as an internal control. (**c**) Quantitative analysis of protein levels from immunoblots. The IFT80 protein levels were normalized to GAPDH. (**d**) Immunofluorescence of cilia in SGC-7901 cells. Cells are stained with anti-acetylated tubulin antibody (green) and anti-IFT80 antibody (red), and nuclei were marked with DAPI (blue) (400×). (**e**) The cilia length was analyzed as shown in (**d**). (**f**) The overexpression of IFT80 was assessed by immunofluorescence. Cells are stained with anti-IFT80 antibody (red), and nuclei were marked with DAPI (blue) (200×). (**g**) Quantification of the fluorescence intensity indicated in (**f**). Data represent the mean ± SEM. *N* = 6. * *P* < 0.05, ** *P* < 0.01, control vs. overexpression of ift80.

The primary cilium is an organelle present in almost all eukaryotic cells, and IFT proteins are required for primary cilia biogenesis. To determine whether cilia are present in the gastric cancer cell line SGC-7901, we constructed the human plasmid *ift80*, and transfected it into the SGC-7901 cell line. IFT80 expression was verified by qRT-PCR and Western blot analysis, and there was a significant increase in both the IFT80 mRNA levels (Figure 2a) and protein levels (Figure 2b,c). To further assess the IFT80 overexpression effects on cilia formation in SGC-7901 cells, the cells were co-stained with antibodies to IFT80 (red), acetylated-tubulin (green), and DAPI (blue) for detecting cilia. As shown in Figure 2d, ift80 is located on the acetylated tubulin of the cilia. Moreover, overexpression of IFT80 increases the length of cilia. Normal cilia length is 1 ± 0.23 μm, however, the observed cilia length is about 4 ± 0.35 μm in SGC-7901 cells overexpressing IFT80 (Figure 2d,e). As visualized by the localization of IFT80, about 15–20% of the control cells showed apparent IFT80 (Figure 2f), while about 35–40% of the IFT80-overexpressed cells expressed IFT80 (Figure 2f,g). These results suggested that overexpression of IFT80 increased cilia formation.

### 2.3. Overexpression of IFT80 Increases the Proliferation and Invasion Potential of SGC-7901 Cells, but Inhibits Apoptosis

**Figure 3:**
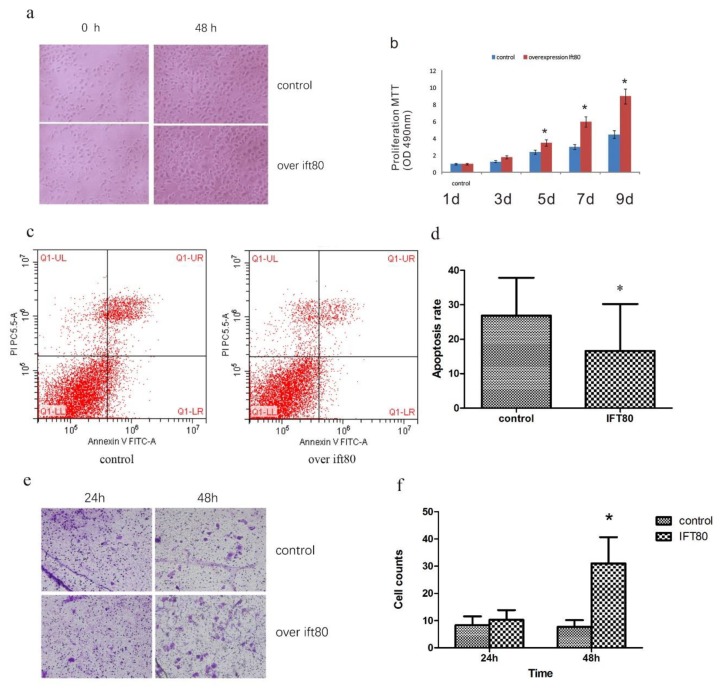
Overexpression of IFT80 enhances the proliferation and invasion potential of SGC-7901 cells, and inhibits apoptosis. (**a**) Top panel, images represent transfection control of the empty vector, 0 h and 48 h. Bottom panel, SGC-7901, the gastric cancer cells transfected with the IFT80 plasmid at 0 h and 48 h (200×). (**b**) Cell proliferation is determined by the CCK-8 assay (*N* = 3, * *P* < 0.05, control vs. overexpression of ift80). (**c**) Flow cytometric analysis of apoptotic cells using the annexin V-FITC reagent. Overexpression of IFT80 inhibits apoptosis in SGC-7901 cells. (**d**) Quantification of images shown in (**c**). (**e**) Transwell migration assay analysis the of invasion ability of SGC-7901 gastric cancer cells. IFT80 overexpression promotes the invasion ability of SGC-7901 cells (200×). (**f**) Quantification of images shown in e. (*N* = 3, * *P* < 0.05, control vs. overexpression of ift80).

To test the effect of IFT80 on the proliferation of the SGC-7901 cell line, we performed an MTT experiment using the CCK-8 assay. The results showed that IFT80 overexpression significantly enhanced cell proliferation (Figure 3a,b). In addition, we evaluated apoptosis in cells overexpressing IFT80. Apoptotic cells were collected and washed with PBS prior to being identified by annexin V staining and detected by flow cytometry. A decrease in apoptotic cells was observed in IFT80-overexpressing cells (apoptosis was 15% ± 10% of cells) compared to control cells (apoptosis was 26% ± 8% of cells) (Figure 3c,d). To detect whether IFT80 enhances the invasion ability of SGC-7901 cells, we performed a Matrigel invasion assay. SGC-7901 cells (2 × 10^4^ cells/well) were seeded in a Matrigel-precoated invasion chamber and incubated at 37 °C for 24 and 48 h. SGC-7901 cells that had migrated from the upper side of the filter to the lower side were counted under a light microscope at a magnification of 200×. By comparing the number of migrating cells, the results demonstrated that overexpression of IFT80 promoted the invasion ability of SGC-7901 cells (Figure 3e,f). These results provide the first evidence that overexpression of IFT80 increases proliferation and invasion capabilities, and decreases apoptosis in the SGC-7901 cell line.

### 2.4. Overexpression of IFT80 Increases the mRNA and Protein Expression of p75NGFR and MMP9 in SGC-7901 Gastric Cancer Cells

**Figure 4:**
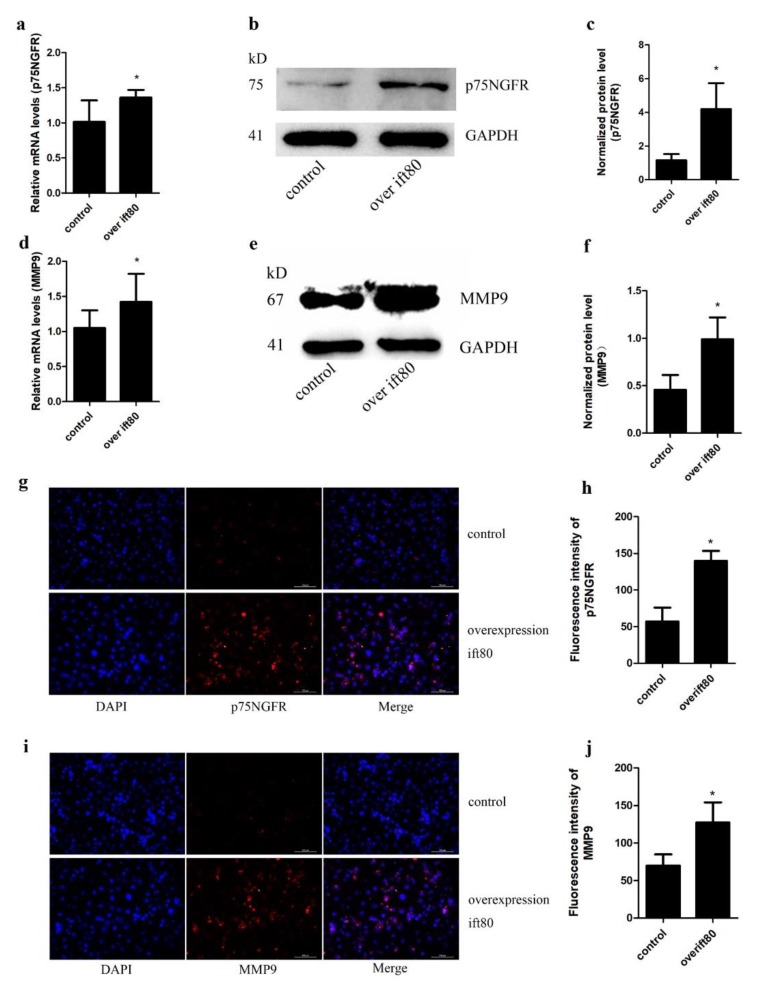
Increased IFT80 expression increases the protein expression levels of p75NGFR and MMP9 in SGC-7901 cells. (**a**) qRT-PCR analysis of p75NGFR expression in control cells and IFT80-overexpressing cells. The expression of p75NGFR is normalized to GAPDH expression. (**b**) The expression of p75NGFR was evaluated by Western blot analysis. GAPDH was used as an internal control. (**c**) Quantitative analysis of p75NGFR protein levels from immunoblots in (**b**). The protein levels of p75NGFR were normalized to GAPDH. (**d**) qRT-PCR analysis of MMP9 expression in control cells and IFT80-overexpressed cells. (**e**) The MMP9 protein expression was assessed by Western blot analysis. (**f**) Quantitative analysis of MMP9 protein levels from immunoblots in e. The protein levels of MMP9 were normalized to GAPDH. (**g**) The expression of p75NGFR was assessed by immunofluorescence in control cells and IFT80-overexpressed cells. Cells are stained with anti-p75NGFR antibody (red) and nuclei were marked with DAPI (blue) (200×). (**h**) Quantification of the fluorescence intensity shown in (**g**). Data represent the mean ± SEM. *N* = 3. * *P* < 0.05, control vs. overexpression of ift80. (**i**). The expression of MMP9 was evaluated by immunofluorescence in control cells and IFT80-overexpressed cells. Cells are stained with anti-MMP9 antibody (red) and nuclei were marked with DAPI (blue) (200×). (**j**). Quantification of the fluorescence intensity shown in (**i**). (*N* = 3, * *P* < 0.05, control vs. overexpression of ift80).

To further study the mechanism by which IFT80 promotes gastric cancer cell invasion, we next examined whether the overexpression of IFT80-enhanced invasion ability of SGC-7901 cells is associated with p75NGFR and MMP9. At both the mRNA (Figure 4a,d) and protein levels (Figure 4b,c,e,f), the expression of p75NGFR and MMP9 were significantly increased in IFT80-overexpressing cells, compared to control cells. In addition, we further confirmed, with immunofluorescence, that the overexpression of IFT80 increased p75NGFR protein expression (Figure 4g,h) and MMP9 protein expression (Figure 4i,j). These results suggested that overexpression of IFT80 enhanced the invasion ability of SGC-7901 cells through increasing the levels of p75NGFR and MMP9.

### 2.5. PD90780, a p75NGFR Antagonist, Inhibits the Increased Invasion Ability Resulting from Overexpression IFT80 in SGC-7901 Gastric Cancer Cells

**Figure 5:**
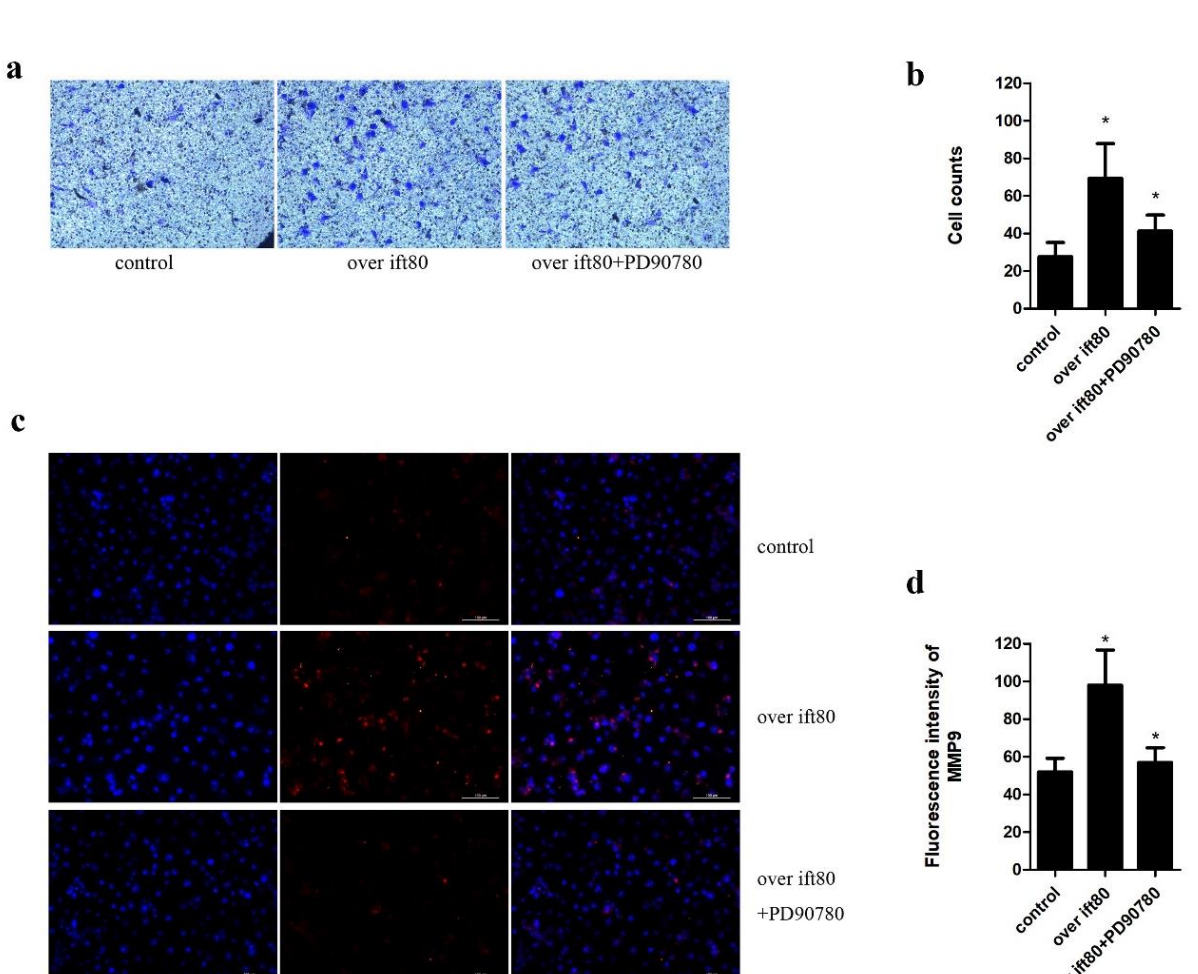
p75NGFR antagonist PD90780 significantly repressed the number of invasive SGC-7901 cells and the protein expression of MMP9. (**a**) Cell invasion was detected in SGC-7901 cells treated with or without PD90780, followed by transfection with the *ift80* plasmid or empty vector (200×). (**b**) Quantification of images shown in a. Data points indicate the mean, while the error bars represent the SEM. Data were analyzed using the two-way ANOVA followed by Tukey’s multiple comparison test. * *P* < 0.05. (**c**) Immunofluorescence staining for MMP9 location and expression with MMP9 antibody (red). Nuclei were stained with DAPI (blue) (200×). (**d**) Quantification of the fluorescence intensity shown in (**c**). Data points indicate the mean, while error bars represent the SEM. Data were analyzed using the two-way ANOVA followed by Tukey’s multiple comparison test. * *P* < 0.05 (200×).

Extracellular matrix degradation is an indispensable step in tumor metastasis and invasion, which is mainly mediated by the balance between selected MMPs, such as MMP9 and MMP2 [27,28]. To further confirm the role of p75NGFR in the regulation of expression of MMP9, we used PD90780, which is a p75NGFR antagonist. In the Matrigel invasion assay, treatment with PD90780 inhibits the invasion ability of SGC7901 cell line when compared to cells overexpressing IFT80 (Figure 5a,b). In addition, our data confirmed that the expression of MMP9 in SGC-7901 cells could be downregulated by a p75NGFR antagonist (Figure 5c,d). Taken together, these results suggested that the reduction in MMP9 protein expression and inhibition of the metastatic ability of gastric cancer cells was at least partially mediated by the downregulation of p75NGFR.

## 3. Discussion

IFT80 partial mutations in humans cause JATD and SRP syndrome type III. Both diseases are characterized by bone abnormalities, including constriction of the ribcage, polydactyly, and shortening of the long bones [16,17,18,19]. Conditional knockout of the IFT80 protein in animal experiments revealed Hedgehog signaling similar to the occurrence and defects of the malformations of JATD and SRPIII [16]. In addition, studies have also found that IFT80 affects the development of photoreceptors [29]. Defects in the IFT protein can cause abnormalities in Hedgehog signaling. It has been found that IFT80 plays an important role in the process of bone formation by regulation of the Hedgehog signaling pathway, and affects the differentiation of cartilage by regulating the Hedgehog and Wnt signaling pathways [30], which shows that it plays a critical role in the process of cell differentiation. Abnormal cell differentiation forms cancerous cells which, in turn, cause cancer. Until now, whether ift80 was expressed in gastric cancer cells and the mechanism of progress of gastric cancer have largely been unknown. 

Cilia are microtubule-based organelles found on most eukaryotic cells [31]. The IFT proteins are key proteins for cilia formation, and it seems that this organelle has developed into a complex signaling center in mammals, providing important sensory functions [32,33]. The primary cilium also plays a critical role in cell-to-cell communication by sensing extracellular signals during development. Two extensively studied pathways, where in vivo and in vitro data suggest a role for cilia, are the Hedgehog (Hh) and Wnt pathways [8]. At the molecular level, the Hedgehog signaling pathway has been found to be abnormally activated in various types of cancers, and it has also been shown that the IFT protein is associated with cancer. Therefore, in this study, our aim was to investigate the effects of IFT80 overexpression on the biological functions of gastric cancer, in which the invasion potential of cancer cells is essential for cancer metastasis. Our study has also shown that IFT80 was overexpressed in gastric cancer, and that overexpression of IFT80 increases the proliferation of SGC-7901 cells, but inhibits apoptosis.

Nerve growth factor (NGF) supports neuronal differentiation and survival [34], and is also considered a regulatory cytokine for lymphoid cell proliferation and differentiation [35,36,37,38]. It has been reported that low levels of NGF are expressed in normal peripheral nerves, but a rapid increase occurs after experimental nerve injury [39]. In addition, the expression of p75NGFR has been observed in several human cancers, such as thyroid carcinoma, liver cancer, and stomach cancer [40,41,42,43]. Previous studies have suggested that NGF and its receptors are involved in thymus stroma organogenesis and proliferation [44]. MMP9 is an endopeptidase responsible for the degradation of extracellular matrix proteins, and plays a role in many physiological processes, including development, tissue repair, and remodeling. High levels of MMPs are observed in the pathology of major inflammation and cancer [45]. Since MMP9 plays an important role in cell proliferation, invasion, and migration, it can thereby participate in the process of gastric cancer metastasis [46]. This study detected that the overexpression of IFT80 increased the expression of p75NGFR and MMP9 in SGC-7901 gastric cancer cells, indicating that IFT80 overexpression increased the invasion of gastric cancer. Further, we investigated the possible role of p75NGFR in MMP9-related invasion by treating SGC-7901 cells with the p75NGFR antagonist PD90780, before carrying out the invasion assay. The results showed that treatment with PD90780 could inhibit the invasion ability of SGC-7901 cells. In addition, we found that downregulation of p75NGFR with PD90780 could reduce the expression of MMP9 in SGC-7901 cells. In short, these data suggest that the reduction in MMP9 protein expression and inhibition of gastric cancer cell metastasis was at least partially mediated by the downregulation of p75NGFR. Moreover, overexpression of IFT80 enhances the proliferation and invasion potential of MKN-45 cells, which can be inhibited by the p75NGFR antagonist PD90780. Cells overexpressing IFT80 demonstrated that IFT80 significantly enhances cell proliferation. (Figure S1c). MKN-45 gastric carcinoma cells were allowed to migrate through Matrigel-coated Transwell inserts into lower wells, and it was determined that overexpression of IFT80 promotes the invasion of MKN-45 cells, but treatment with PD90780 inhibits the invasion ability (Figure S1a,b). In addition, we measured the protein levels of IFT80, MMP9, and p75NGFR. (Figure S1d–g). Our data confirmed that the expression of all three were significantly increased in IFT80-overexpressing cells; however, the expression of p75NGFR and MMP9, in MKN-45 cells, was reduced by treatment with p75NGFR antagonist PD90780. These data suggest that the reduction in MMP9 protein expression and metastatic inhibition of gastric cancer cells was at least partially mediated by the downregulation of p75NGFR.

Here, we present the first evidence that IFT80 is significantly highly expressed in gastric cancer cells, and we also show that IFT80 increases the invasion and metastasis of gastric cancer by the upregulation of p75NGFR and MMP-9. Although the regulation of IFT80 has mainly been studied in osteoblasts [20], zebrafish photoreceptor cell death [29], mouse multifinger syndrome [16], and chondrocyte differentiation regulation [31], this report is the first to demonstrate the role of IFT80 in gastric cancer.

## 4. Materials and Methods

### 4.1. Reagents and Chemicals

Dimethyl sulfoxide (DMSO) and PD90780 were purchased from Sigma-Aldrich (St. Louis, MO, USA). 

### 4.2. Clinical Specimens Collected and Immunohistochemistry

Forty-eight patients with gastric carcinoma were enrolled in the study. This study was approved by the Medical Research Ethics Committee of Chongqing Medical University (20 January 2012, Chongqing, China). Retrieved tissues were fixed, decalcified in 10% formalin, and embedded in paraffin 24 h posttreatment. The fixed gastric cancer tissues were blocked and incubated with IFT80 antibody (1:1000, 16643-1-AP, Abcam, Cambridge, MA, USA). After being washed, tissues were incubated with a biotin-labeled secondary antibody for 30 min, followed by incubation with streptavidin-HRP conjugate for 20 min at RT. The presence of the target protein was visualized by DAB staining and examined under a microscope. Stains with control IgG were used as negative controls.

### 4.3. Cell Culture and Treatment

The human gastric cancer cell lines SGC-7901 and MKN-45 (ATCC, Manassas, VA, USA) were transfected with either the control or IFT80 plasmid, and the expression of IFT80 was confirmed by Western blot analysis. Cells were grown in Dulbecco’s Modified Eagle Medium (Gibico, Grand Island, NY, USA) with 10% fetal bovine serum and antibiotics (100 units/mL penicillin and 100 μg/mL streptomycin), added at 37 °C in an atmosphere of 5% CO_2_ in air.

### 4.4. Apoptosis Assays

We evaluated the effect of IFT80 overexpression on apoptosis. Cells were co-stained with annexin V and PI, and subjected to flow cytometry analysis following the manufacturer’s instructions.

### 4.5. Transwell Invasion Assays

The effect of IFT80 overexpression on cell invasion was measured in a Transwell chamber containing a filter applied to the upper surface with a basement membrane (BD Falcon, Bedford, MA, USA). Cells were seeded on filters coated with Matrigel. Culture media supplemented with 1% FBS was added to both chambers to measure the invasion due to the overexpression of IFT80. Cells were incubated at 37 °C for 24 or 48 h, to allow the Matrigel to adhere to the opposite surface of the filter. Cells on the upper side of the filter were mechanically removed, and the filter was fixed with 100% methanol and stained with Giemsa. The invasion ability was determined by the number of penetrating cells under a microscope at 200× magnification using 5 random fields in each well. Each experiment was done in triplicate.

### 4.6. Immunofluorescence

Immunofluorescence staining analysis of IFT80 expression in mouse tissues was performed as described previously [12]. The cells were seeded at 4 × 10^4^ cells/well on 24-well plates, and incubated overnight, followed by infection with the IFT80 and/or control plasmid for 24 h. The cells were sequentially induced with media containing 10% FBS for 3 days. After 48 h of serum starvation (without FBS), the cells were fixed with 100% ice-cold methanol for 10 min, and washed 3 times with PBS. Fixed cells were blocked with 5% BSA in PBS for 60 min. The cells were then incubated with primary antibody diluted in PBS containing 1% BSA for 1 h. The primary antibodies used were as follows: anti-IFT80 antibody (Abnova, Walnut, CA, USA; 1:500) and mouse monoclonal anti-acetylated tubulin antibody (Sigma, T7451, 6-11B-1, 1:1,000). After washing 3 times with PBS, Alexa Fluor 488- and Alexa Fluor 568 (Invitrogen, Grand Island, NY, USA)-conjugated anti-rabbit or anti-mouse IgG was added in PBS with 1% BSA for 1 h. In the final washes, 6-diamidino-2-phenylindole (DAPI) (Sigma) was added, and used as a counterstain for nuclei. Fluorescence images were acquired using a Zeiss Axio Imager microscope.

### 4.7. qRT-PCR

Total RNA was extracted from cells using TRIzol reagent (Invitrogen, Carlsbad, CA, USA) following the manufacturer’s instructions. cDNA was synthesized from 3 mg total RNA by the RNA to cDNA EcoDry Premix kit (Clontech, Palo Alto, CA, USA). Quantitative real time PCR (qRT-PCR) experiments were performed with the Bio-Rad MJ Mini Option Real-Time PCR System in triplicate, and data analysis was carried out using CFX Manager Software version 1.5. The PCR data were normalized to GAPDH expression. The sequences of each primer pair are as follows: GAPDH (sense: GACCACAGTCCATGCCATCA, antisense: GTCAAAGGTGGAGGAGTGGG); p75NGFR (sense: CCGTTGGATTACACGGTCCA, antisense: GTTCTGCTTGCAGCTGTTCC); IFT80 (sense: AGCAGGCAAGCAGCTAATCA, antisense: CTCCAGCAATCTGAGTGCCA); MMP9 (sense: CATCCGGCACCTCTATGGTC, antisense: GCTCCTCAAAGACCGAGTCC). All reactions were run in triplicate, and normalized to the housekeeping gene GAPDH. Calculations were performed according to the double delta Ct method.

### 4.8. Western Blot

Protein concentration was measured using the BCA protein assay reagent (Pierce, Rochford, IL, USA). Equal amounts of protein were denatured in SDS and separated on 10% SDS–PAGE gels. Proteins were transferred to polyvinylidene difluoride membranes in buffer containing 25 mM Tris, 192 mM glycine and 20% methanol. The membranes were blocked with 5% milk, incubated with primary antibody overnight at 4 °C, and then incubated with horseradish peroxidase (HRP)-conjugated goat anti-rabbit IgG antibody (1:10,000, A-11034, Novex, Carlsbad, CA, USA) at room temperature for 1 h. Visualization was performed with Western Bright ECL HRP (Advansta, San Jose, CA, USA). GAPDH (1:2,000, A00191, Genscript, Piscataway, NJ, USA) was used as the internal control. The primary antibodies used were as follows: anti-MMP9 antibody (1:1000, 10375-2-AP, Proteintech, Rosemount, MN, USA) and anti-p75NGFR antibody (1:1000, YT3116, ImmunoWay, Plano, TX, USA).

### 4.9. Statistical Analysis

All data are presented as mean ± SEM. (*N* ≥ 3). Comparison between two groups used the Student’s *t*-test and grouped samples underwent analysis by two-way ANOVA, followed by Tukey’s multiple comparison test. *P* < 0.05 was considered to be significant. GraphPad Prism (GraphPad Software, Inc., San Diego, CA, USA) was used for these analyses.

## Supplementary Materials

Supplementary materials can be found at http://www.mdpi.com/1422-0067/19/11/3616/s1.
