# Supplementary material for: IFT80 Improves Invasion Ability in Gastric Cancer Cell Line via ift80/p75NGFR/MMP9 Signaling
Source: Int J Mol Sci. 2018 Nov 16;19(11):3616. doi: 10.3390/ijms19113616 (PMC6274718; doi:10.3390/ijms19113616)
Supplement: Supplementary file 1 [file ijms-19-03616-s001.pdf]

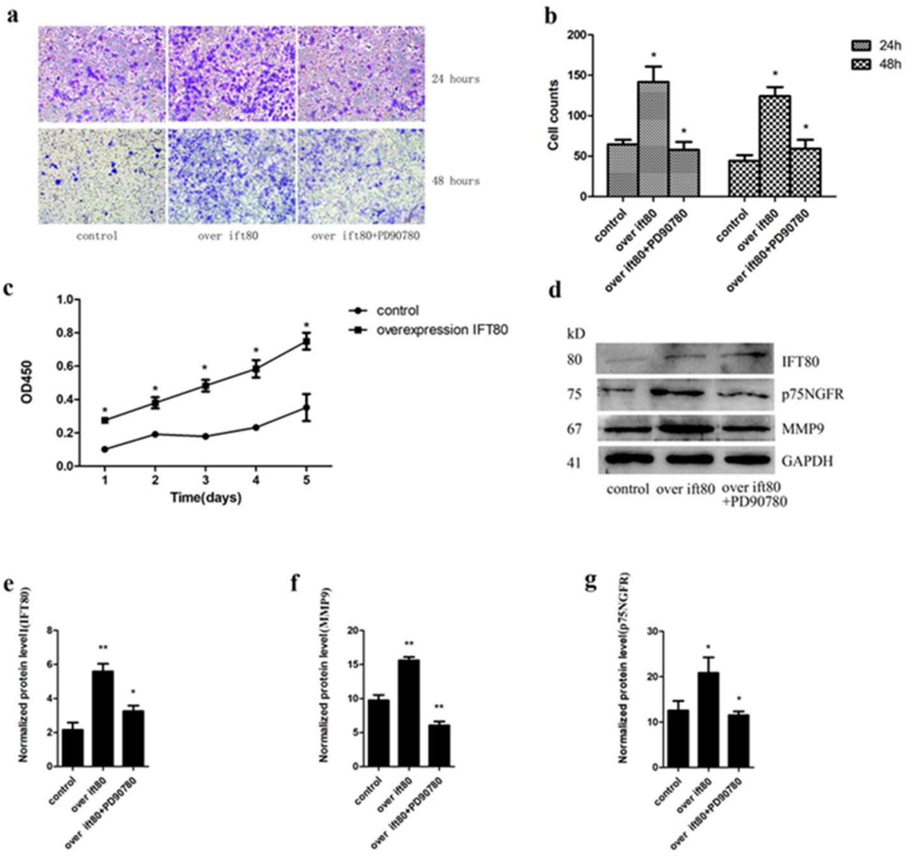

Supplementary Figure. 1

Supplementary Figure. S1. Overexpression of IFT80 enhances the proliferation and invasion potential of MKN-45 cells, but p75NGFR antagonist PD90780 significantly decreases the number of invasive MKN-45 cells and the expression of the proteins p75NGFR and MMP9. (a). Cell invasion was evaluated in MKN-45 cells treated with or without PD90780, followed by transfection with the IFT80 plasmid or empty vector (200×). (b). Quantification of images as shown in a. Data points indicate the mean, while error bars represent the SEM. Data were analyzed using the two-way ANOVA followed by Tukey's multiple comparison test. *$P<0.05$. (c). Cell proliferation is assessed by the CCK-8 assay. Data points indicate the mean, while error bars represent the SEM. Data were analyzed using the two-way ANOVA followed by Tukey's multiple comparison test. *$P<0.05$. (d). The expression of IFT80, MMP9 and p75NGFR were assessed by Western blot analysis. (e). Quantitative analysis of IFT80 protein levels from immunoblots as in d. The protein levels of IFT80 were normalized to GAPDH. Data points indicate the mean, while error bars represent the SEM. Data were analyzed using the two-way ANOVA followed by Tukey's multiple comparison test. *$P<0.05$. (f). Quantitative analysis of MMP9 protein levels from immunoblots as in d. The protein levels of MMP9 were normalized to GAPDH. Data points indicate the mean, while error bars represent the SEM. Data were analyzed using the two-way ANOVA followed by Tukey's multiple comparison test. *$P<0.05$. (g). Quantitative analysis of p75NGFR protein levels from immunoblots as in d. The protein levels of p75NGFR were normalized to GAPDH. Data points indicate the mean, while error bars represent the SEM. Data were analyzed using the two-way ANOVA followed by Tukey's multiple comparison test. *$P<0.05$. **$P<0.01$.
